# Temporal trends in lung cancer mortality and years of life lost in Wuhan, China, 2010-2019

**DOI:** 10.3389/fonc.2022.1030684

**Published:** 2022-11-15

**Authors:** Yaqiong Yan, Yudiyang Ma, Yimeng Li, Xiaoxia Zhang, Yuanyuan Zhao, Niannian Yang, Chuanhua Yu

**Affiliations:** ^1^ Wuhan Center for Disease Control and Prevention, Wuhan, Hubei, China; ^2^ Department of Epidemiology and Biostatistics, School of Public Health, Wuhan University, Wuhan, China; ^3^ Department of Chronic Disease Epidemiology, School of Public Health, Yale University, New Haven, CT, United States

**Keywords:** lung cancer, mortality, years of life lost, temporal trends, burden of disease

## Abstract

**Objective:**

Lung cancer is responsible for millions of deaths yearly, and its burden is severe worldwide. This study aimed to investigate the burden of lung cancer in the population of Wuhan based on the surveillance data from 2010 to 2019.

**Methods:**

Data of this study was obtained from the Mortality Register System established by the Wuhan Center for Disease Control and Prevention. The study systematically analyzed the burden of lung cancer deaths in the population of Wuhan and its 13 administrative regions from 2010 to 2019 *via* the Joinpoint regression models, Age-Period-Cohort (APC) models, and decomposition analysis.

**Results:**

This study found the upward and downward trends in the age-standardized mortality rates (ASMRs) and age-standardized years of life lost rates (ASYLLRs) of lung cancer from 2010 to 2019. In Joinpoint regression models, the corresponding estimated annual percentage change (EAPC) were 1.00% and -1.90%, 0.60%, and -3.00%, respectively. In APC models, lung cancer mortality tended to increase with age for both sexes in Wuhan, peaking at the 85-89 age group; The period effects for different populations have started to gradually decline in recent years. In addition, the cohort effects indicated that the risk of lung cancer death was highest among those born in the 1950s-1955s, at 1.08 (males) and 1.01 (females). Among all administrative districts in Wuhan, the ASMR of lung cancer in the Xinzhou District has remained the highest over the study period. In decomposition analysis, both population aging (*P*<0.01) and population growth (*P*<0.01) aggravated (Z>0) lung cancer deaths in the Wuhan population.

**Conclusions:**

The burden of lung cancer death in the Wuhan population has shown a gradual decline in recent years, but the impact of aging and population growth on lung cancer mortality should not be ignored. Therefore, lung cancer surveillance must be strengthened to reduce the burden of lung cancer in Wuhan.

## Introduction

As a multi-stage and multi-factor cancer, lung cancer is the leading cause of cancer-related death in China, especially for males (1). Due to the poor prognosis and high patient mortality rate, lung cancer also proves to be the leading cause of cancer-related death globally (2). In China, the mortality rate of lung cancer has increased about four times over the past decades, and deaths caused by lung cancer account for 27.3% of all cancer-related deaths in 2020 (3, 4). In recent years, lung cancer has replaced stomach cancer as the leading cause of cancer death. China is the most populous country in the world, and the disease burden of lung cancer varies among populations living in different regions of China because of the vast geographical area (5). According to the global limitation of disease study 2019, the age-standardized mortality rates (ASMRs) rose from 31.18/100,000 in 1990 to 38.70/100,000 in 2019, much higher than the global average level (6). Therefore, the lung cancer epidemic poses a severe health burden to the Chinese population.

Wuhan, located in Hubei province, is the largest city in central China. As a highly developed metropolis in China, Wuhan has a large population and a prosperous economy. Being the first leading cause of death in Wuhan, lung cancer represents a significant challenge to public health in Wuhan (7). Lung cancer is related to diverse factors, such as tobacco exposure, indoor and outdoor air pollution, poor dietary habits, occupational exposure, previous chronic lung infections (tuberculosis or bronchial infections), etc. (8, 9). Most of the current studies were conducted at the national level, but few of them focused on the lung cancer burden at the provincial or municipal level. The temporal trend of lung cancer deaths in Wuhan could reflect the movement and variations in the population of Hubei province and other cities in Central China.

This study aimed to explore the temporal trends of lung cancer mortality by sex and administrative regions over the last decade in Wuhan, with an emphasis on decomposing the contributions of demographic factors and investigating the detached effects of age, period, and cohort. Furthermore, this study could also shed light on priorities that deserve policymakers’ attention for targeted interventions by comparing discrepancies in lung cancer burden between the central and surrounding urban areas.

## Method

### Data sources

Data for this study was derived from the Mortality Register System established by the Wuhan Center for Disease Control and Prevention (CDC). We included all death cases of lung cancer in Wuhan recorded between Jan 1, 2010, and Dec 31, 2019. The death cases were classified according to the International Statistical Classification of Diseases 10th Revision (ICD-10: C33, C34-C34.92, D02.1-D02.3, D14.2-D14.3, D38.1, Z12.2, Z80.1-Z80.2, and Z85.1-Z85.20). Demographic data consisted of information on age, sex, date of death, and cause of death were also included in our analysis. Annual population data for the whole study period were obtained from the Wuhan Public Security Bureau. The reason for data in this study was surveillance data. The informed consent was unnecessary. In addition, this study was approved by the Ethical Committee of the Wuhan CDC and was conducted in compliance with the tenets of the Declaration of Helsinki. The results were reported under the STORBE statement.

There are 13 administrative districts in Wuhan, of which Jiang’an District, Jianghan District, Qiaokou District, Hanyang District, Wuchang District, Qingshan District, and Hongshan District are the central urban areas, and Dongxihu District, Hannan District, Caidian District, Jiangxia District, Huangpi District, and Xinzhou District are the surrounding metropolitan areas.

### Statistical analysis

Death cases were directly counted according to the origin data. The years of life lost (YLLs) was an index representing premature death in the population. It was estimated by summing up the remaining life expectancy for people dying in each age group (10). The reference life expectancy was 86.6 years, derived from the first age group (0-4 age group) in the standardized life expectancy table in the global burden of disease study 2016 (11). Meanwhile, we use the data obtained from the sixth Chinese census (http://www.stats.gov.cn/tjsj/pcsj/rkpc/6rp/indexch.htm) as the standard population. This study then calculated the mortality rate, ASMRs, years of life lost rates (YLLRs), and age-standardized years of life lost rates (ASYLLRs) by age groups, sex, and administrative regions.

In the Joinpoint model, the estimated annual percent changes (EAPCs) and the average annual percent changes (AAPCs) were calculated to depict the temporal trends of the age-standardized rates (ASRs) (12). If the lower boundary of the EAPCs’ 95% confidence intervals (CIs) were higher than 0, the ASRs were deemed to keep increasing during the study period. While the higher boundary of the EAPCs’ 95% CIs was lower than 0, the ASRs were considered to decline (13).

A latest developed decomposition method was performed to explore the attributable demographic factors (population growth, population aging, and changes in age-specific mortality in lung cancer), which drove the changes in lung cancer deaths in Wuhan from 2010 to 2019 (14). This method has considered the two-way and three-way interactions between the mentioned demographic factors. The influence of these factors on the changes in lung cancer deaths in Wuhan was presented by the absolute and relative contributions. The real contribution was the total of lung cancer deaths attributed to each mentioned demographic factor. At the same time, the relative contribution was the absolute contribution divided by the total lung cancer deaths. Furthermore, we detected the monotonic trends of the absolute or relative contributions during 2010-2019 in Wuhan *via* the Mann-Kendall monotonic trend test (14). A positive Z value indicates a monotonic increasing trend in the whole or relative contributions. In contrast, a negative Z value means a monotonic decreasing trend in the absolute or relative contributions.

The age-period-cohort (APC) model could decompose the risks of death that are experienced by individuals in the current year and the accumulation of health risks since birth (15). To fit the APC model, death cases of lung cancer between 20-89 years old were divided into 12 consecutive 5- year age groups (death cases below 20 years old were excluded due to few people dying younger than 20). The study period was arranged into two consecutive 5- years period groups and 15 successive 5- years cohort groups. For dealing with the “non-identifiable problem” in the APC model, this study fitted a sequence of models, such as the one-factor age model, the two-factor age-drift (Ad), age-period (AP) and age-cohort (AC) models, and the full three-factor APC model (16). The statistical significances of different terms added models were tested. We selected the best-fitting model by comparing the differences in model deviances and with the degree of freedom *via* the Chi-square test (17).

The detailed information about the models used in analyses in Supplementary Material. All analyses in this study proceeded in R software (version 4.0.1, package: epitools (0.5-10.1), Epi (2.44)) and the Joinpoint regression program (version 4.8.0.1). Two-tailed tests were performed to determine all *P* values, and *P* less than 0.05 is considered statistically significant.

## Results

### The temporal trends of lung cancer deaths in Wuhan

Descriptive data with essential characteristics for lung cancer in Wuhan were summarized in **Table 1**. In both males and females, the mortality rate and YLLRs of lung cancer kept increasing during 2010-2019. But after standardization, the ASMRs of lung cancer in the whole population of Wuhan first rose from 48.89/100,000 in 2010 to 52.61/100,000 in 2017, then declined to 50.48/100,000 in 2019. The trend of ASYLLRs corresponds with the trend of ASMRs in the same period. Moreover, there was a significant difference between men and women in ASR of lung cancer (*P*<0.05). Males have experienced a more severe burden of lung cancer death than females in Wuhan over the study period.

**Table 1:** Trends in the burden of lung cancer death in Wuhan, 2010-2019.

Year	Deaths	Mortality (1/100,000)	ASMRs (1/100,000)	YLLs	YLLRs (1/100,000)	ASYLLRs (1/100,000)
Both						
2010	3363	68.86	48.89	83238	1277.14	1199.47
2011	3485	51.60	48.48	84821	1320.41	1225.57
2012	3779	54.24	49.43	89196	1344.70	1222.42
2013	3885	56.96	50.84	90840	1327.59	1185.70
2014	4263	56.78	49.92	100235	1489.50	1258.00
2015	4393	63.35	51.87	102488	1504.20	1237.62
2016	4702	64.48	51.05	107340	1564.94	1255.66
2017	4632	68.54	52.61	104601	1520.26	1194.25
2018	4878	67.32	50.57	107953	1542.96	1172.54
2019	4948	69.73	50.48	109016	1516.92	1130.66
Male						
2010	2488	75.53	72.94	62692	1903.15	1834.15
2011	2591	79.82	76.51	63049	1942.17	1854.98
2012	2767	82.62	77.98	65917	1968.13	1845.66
2013	2889	83.67	78.79	68034	1970.78	1813.81
2014	3180	93.54	80.71	75040	2207.43	1915.86
2015	3235	94.12	80.34	76139	2214.98	1869.14
2016	3478	100.50	81.20	80233	2318.38	1907.91
2017	3423	98.71	78.45	78293	2257.57	1830.15
2018	3610	102.48	78.53	81105	2302.49	1802.22
2019	3649	101.01	75.74	81254	2249.35	1723.33
Female						
2010	875	27.15	23.43	20546	637.41	578.96
2011	893	28.11	24.43	21772	685.20	622.62
2012	1011	30.80	24.91	23279	708.88	627.13
2013	996	29.39	24.46	22806	672.68	587.16
2014	1083	32.52	25.24	25194	756.59	628.58
2015	1158	34.30	25.68	26349	780.48	631.29
2016	1223	36.00	26.09	27107	797.65	632.80
2017	1209	35.42	24.73	26309	770.95	587.57
2018	1269	36.52	24.69	26848	772.83	576.75
2019	1300	36.36	24.30	27762	776.70	572.29

### The Joinpoint regression analysis

By fitting the Joinpoint regression model, a turnaround in the trend of ASMRs or ASYLLRs for lung cancer in the population of Wuhan was observed from 1990 to 2019 (**Table 2**). The EAPCs of ASMRs were 1.00% (0.40%, 1.70%) and -1.90% (-6.40%, 2.80%) in 2010-2017 and 2017-2019, respectively. Yet, only the upward trend between 2010-2017 was statistically significant (*P*<0.05). The upward and downward trends in both males (1.90% in 2010-2015, -1.70% in 2015-2019) and females (1.40% in 2010-2016, -2.30% in 2016-2019) were statistically significant (*P*<0.05). In terms of ASYLLRs of lung cancer in Wuhan, the EAPCs were 0.60% (-0.50%, 1.80%) and -3.30% (-6.30%, -0.10%) in 2010-2016 and 2016-2019. Among different sex groups, only the downward trend in males (-3.00% in 2016-2019) was statistically significant (*P*<0.05).

**Table 2:** The Joinpoint regression models for ASMRs and YLLRs of cancer in Wuhan, 2010-2019.

	Trend	Year	EAPCs (%, 95% CIs)	*P* value
ASMRs				
Both	Trend 1	2010-2017	1.00 (0.40, 1.70) *	<0.01^*^
Both	Trend 2	2017-2019	-1.90 (-6.40, 2.80)	0.30
Male	Trend 1	2010-2015	1.90 (1.00, 2.90) *	<0.01^*^
Male	Trend 2	2015-2019	-1.70 (-2.90, -0.50) *	<0.01^*^
Female	Trend 1	2010-2016	1.40 (0.60, 2.20) *	<0.01^*^
Female	Trend 2	2016-2019	-2.30 (-4.50, -0.10) *	<0.01^*^
YLLRs				
Both	Trend 1	2010-2016	0.60 (-0.50, 1.80)	0.18
Both	Trend 2	2016-2019	-3.30 (-6.30, -0.10) *	<0.01^*^
Male	Trend 1	2010-2016	0.60 (-0.50, 1.70)	0.21
Male	Trend 2	2016-2019	-3.00 (-5.9, -0.10) *	<0.01^*^
Female	Trend 1	2010-2015	1.30 (-1.70, 4.20)	0.32
Both	Trend 2	2015-2019	-2.70 (-6.40, 1.10)	0.11

*P* in bold represent statistically significance at *P* < 0.05 (*).

CIs denote confidence intervals.

### Decomposition analysis

Decomposition analysis showed that both the population aging and the population growth drove the number of lung cancer deaths in Wuhan. The population aging played the dominant role (Z = 3.94), followed by the population growth (Z = 3.58), but the lung cancer deaths due to the changes in the age-specific mortality rate were insignificant (*P* = 0.11) after the Mann-Kendall monotonic trend test (**Table 3**).

**Table 3:** Contribution of changes in population aging, population growth, and age-specific mortality rate of lung cancer to variations of lung cancer deaths in Wuhan, 2010-2019.

Year	Due to population aging	Due to population growth	Due to age-specific mortality rate	Net change
2010 (reference)	–	–	–	–
2011	29.68	-52.1	141.48	121.53
2012	82.31	68.98	270.45	415.54
2013	131.85	192.98	213.9	521.9
2014	411.1	146.4	367.38	899.98
2015	543.68	209.44	315.5	1030.5
2016	682.75	262.16	451.5	1338.53
2017	750.99	272.1	303.02	1269.04
2018	875.2	375.74	351.33	1515.44
2019	964.82	516.48	219.59	1585.42
*Z* values	3.94	3.58	1.61	3.76
*P* values	<0.01^*^	<0.01^*^	0.11	<0.01^*^

*P* in bold represent statistically significance at *P* < 0.05 (*).

**Figure 1:**
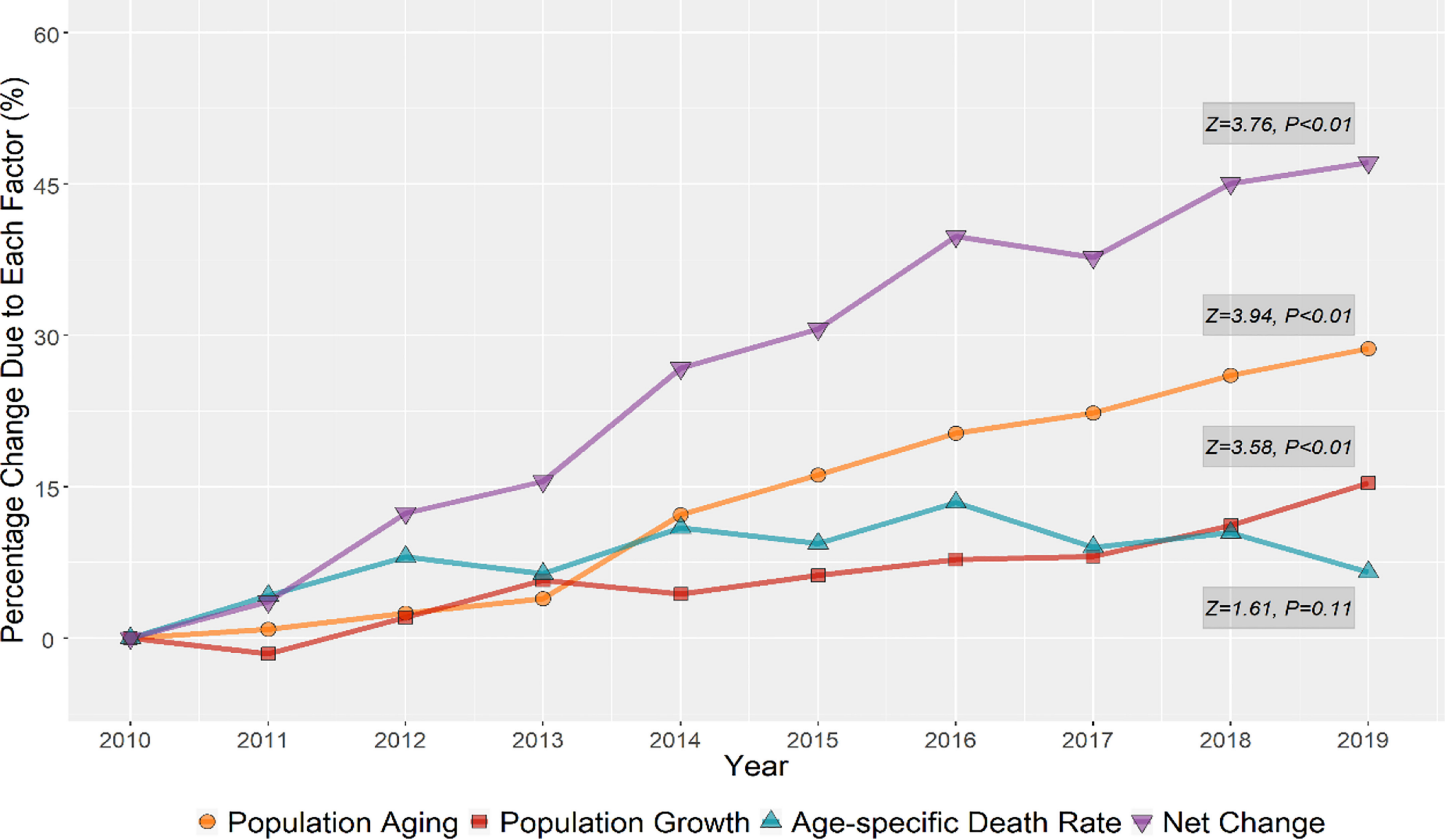
Relative contributions of changes in population aging, population growth, and age-specific lung cancer mortality rate to variations of lung cancer deaths in the population of Wuhan, 2010-2019.

There was an increase of 147.13% (additional 1585 deaths) in lung cancer deaths in Wuhan in 2019 from 2010. According to **Figure 1**, this increase was primarily driven by the population aging (28.69% increase from 2010) and the population growth (15.36% increase from 2010).

We also conducted the decomposition analysis to study the lung cancer deaths influenced by demographic factors in both central and surrounding urban areas of Wuhan (**Table S1**). For lung cancer deaths in the population of central urban areas in Wuhan, the absolute and relative contributions from the population aging (616.07 deaths and 29.19% increase in 2019 compared to 2010) and the population growth (362.83 deaths and 17.19% increase in 2019 compared to 2010) still dominantly affected the increasement of lung cancer deaths. But the relative contribution for the changes in age-specific mortality rate was in decline, with 5.84% reductions in 2019 compared to 2010. For lung cancer deaths in the population of surrounding urban areas in Wuhan, though the contributions from the population aging (360.08 deaths and 28.76% increase in 2019 compared to 2010) and the population growth (153.89 deaths and 12.28% increase in 2019 compared to 2010) kept increase, the changes of age-specific mortality rate became the main demographic factor (789.71 deaths and 25.65% increase in 2019 compared to 2010) driving the increase of lung cancer deaths during the study period. All the monotonic increasing trends of lung cancer deaths due to demographic factors in both central and surrounding urban areas of Wuhan were statistically significant (*P* < 0.05) (**Figure S1**).

### Age-period-cohort model

The goodness of fit for the APC models of lung cancer mortality in Wuhan was summarized in **Table S2**. We selected the best model based on the deviance and *P* value of fitted models (17). Since there is the “non-identifiable problem” in the APC model, we usually fit the AP or AC model first and then fit the remaining cohort or period effects to the residuals. According to **Table S2**, we found that among all the models, the AC-P model may be the most suitable for our data. Therefore, we choose the AC-P model as our final model for analysis.

**Figure 2:**
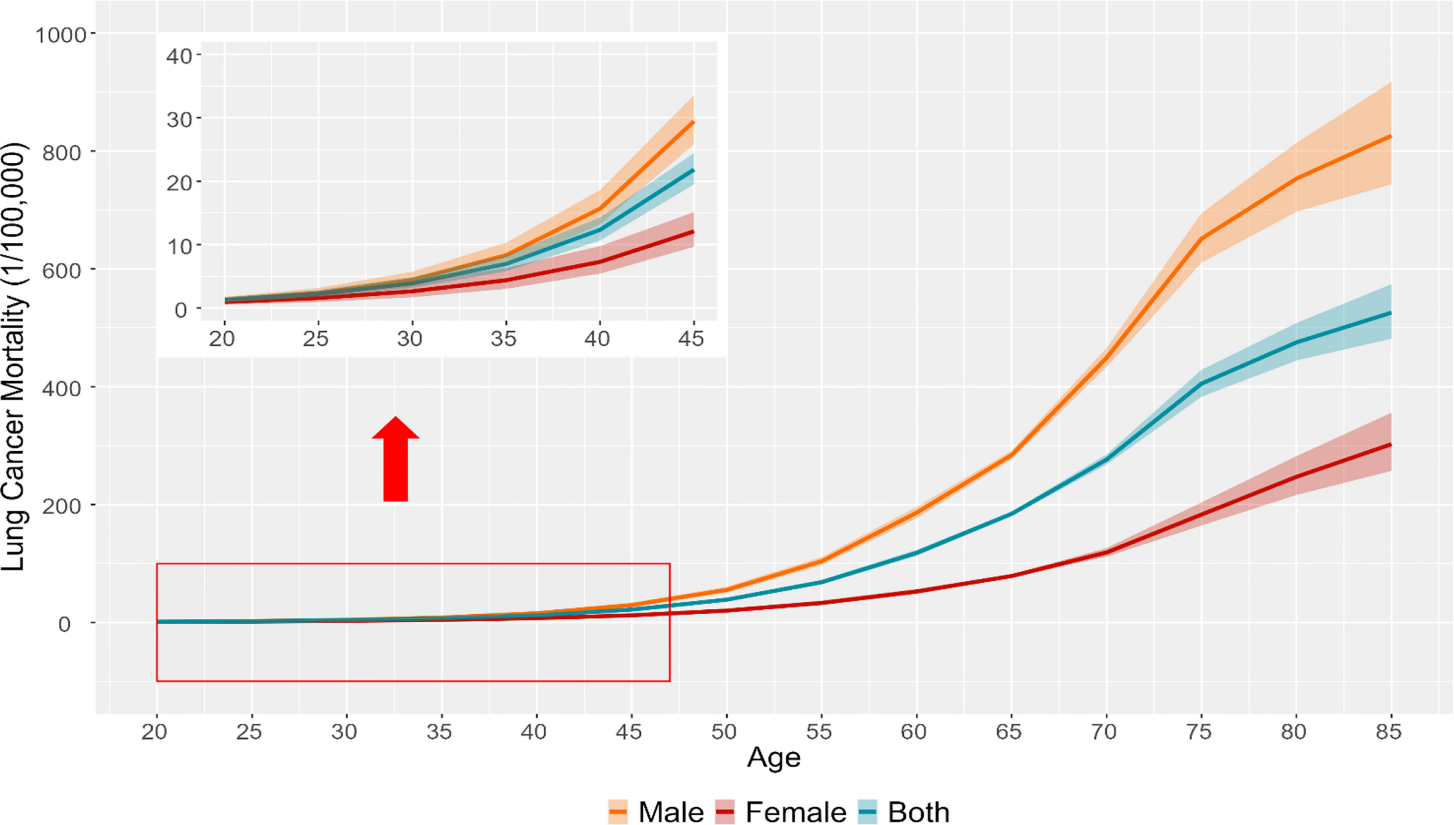
The longitudinal age curves of lung cancer mortality rate and the corresponding 95% CIs for different groups of population in Wuhan (the y-axis for the inside graph was lung cancer mortality, and the x-axis for the inside graph was age).

**Figure 3:**
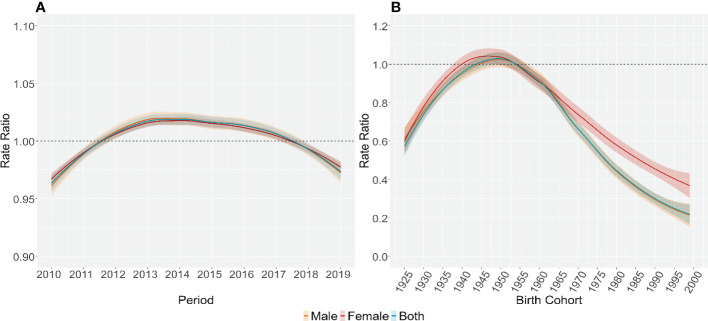
Parameter estimates of period **(A)** and cohort effects **(B)** on lung cancer mortality rate for different groups of population in Wuhan, 2010-2019.

**Figures 2**, **3** illustrated the estimates of age, period, and cohort effects for lung cancer mortality by sex. The age effects escalated exponentially with age and peaked in the 85-89 age group, with males higher than females in the same age group. Throughout the study period, the period effects of lung cancer mortality for different populations in Wuhan showed a trend of increasing and then decreasing, with the period effects for males and females decreasing from 1.02 in 2014 to 0.98 in 2019. For the cohort effects of lung cancer mortality, upward trends were revealed by the model in generations born earlier than 1950s-1955s. While there were reductions in death risk in the cohorts born after 1950s-1955s for both sexes in Wuhan. Compared to those born in the 1950s-1955s, the risk of lung cancer death decreased by 80.42% and 63.40% for males and females born after 1995s, respectively.

### The temporal trends of lung cancer deaths in 13 administrative regions of Wuhan

**Figure 4:**
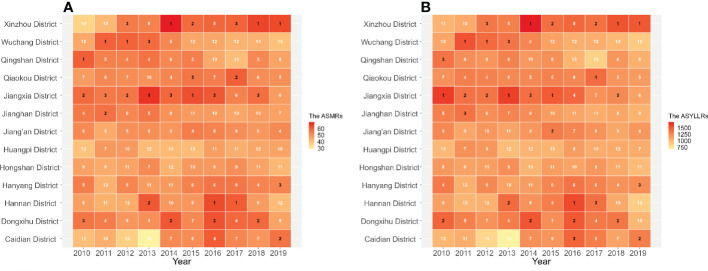
Changes of the ASMRs **(A)** and ASYLLRs **(B)** for lung cancer and the corresponding ranks of the ASRs in the population of 13 administrative regions in Wuhan, 2010-2019.

**Figure 4** demonstrated changes in the ASMRs and ASYLLRs of lung cancer and the corresponding ranks of the ASRs in the population of 13 administrative regions in Wuhan. The ASMRs of lung cancer in the population of Xinzhou District were the highest among all administrative regions over the whole study period, followed by Jiangxia District. Residents in Wuchang District and Hannan District suffered severe death from lung cancer during 2011-2013 or 2016-2017, but the situations been better in recent years. The situations of the ASYLLRs in 13 administrative regions of Wuhan were much like that of the ASMRs.

## Discussion

Our study provided an in-depth insight into temporal trends of lung cancer mortality in Wuhan. There were upward and then downward trends in both ASMRs and ASYLLRs of lung cancer from 2010 to 2019. Among all sociodemographic factors, both the population aging and the population growth could aggravate lung cancer deaths. In the whole Wuhan population, aging was proved to be the most severe influence factor on lung cancer deaths, and the relative contribution increased from 0.88% in 2011 to 28.69% in 2019. Although the changes in age-specific mortality rate have no significant effect on lung cancer deaths in the whole population of Wuhan, its influences on lung cancer deaths in the people of the central urban and surrounding areas presented opposite situations. The results of the APC model showed that after adjusting for the period and cohort effects, lung cancer mortality tended to increase with age for both sexes in Wuhan, peaking at the 85-89 age group. The period effects for different populations have started to gradually decline in recent years. In addition, the cohort effects indicated that the risk of lung cancer death was highest among those born in the 1950s-1955s, at 1.08 (males) and 1.01 (females). The risk of lung cancer death began to decline in subsequent birth cohorts, reaching the lowest level in those born after 1995s. For all administrative districts in Wuhan, the ASMRs and ASYLLR of lung cancer in the Xinzhou District remained the highest over the study period.

The mortality rate of lung cancer has increased in the population of Wuhan from 2010 to 2019. Meanwhile, the mortality was more severe in Wuhan than at the national level in the same period (18). The ASMRs of lung cancer for males in Wuhan were higher than the ASMRs in the Chinese male population, whereas a similar level of lung cancer ASMRs was found in females of Wuhan and China. By the decomposition method, this study discovered that the population aging and the population growth were two main factors contributing to the severe burden of lung cancer deaths in Wuhan. According to previous studies, the average annual growth rate of people aged over 60 years old in Wuhan was 3.00% since Wuhan was listed as a city with an aging population in 1993 (19). By the end of 2017, the number of older adults over 60 in Wuhan had accounted for 20.95% of the total population, which was much higher than the international standard of 10% (20). The large proportion of older adults in the population may lead to a series of problems, such as reduced immunity to disease, lower metabolic levels, or poor nutrition. It is no doubt that the risk of lung cancer death will increase once the elderly population becomes more vulnerable to lung cancer risk factors (air pollution or tobacco exposure, etc.) (21).

Moreover, the age effects that the risk of lung cancer death in the population increases with age in the APC model strengthened findings from the former research, which also confirmed the impact of the population aging on the burden of lung cancer deaths in Wuhan (22–24). The population growth in Wuhan, in addition to driving the population aging, also poses a challenge to the medical system or environmental protection in the city (25). That potential threat might also aggravate the burden of lung cancer deaths in Wuhan.

In the early 2000s, some lung cancer screening studies using low-dose computed tomography (LDCT) were initiated only in some economically developed urban areas or in high-risk rural areas of China (26). The population-based lung cancer screening program using LDCT has been available in the Chinese National Lung Cancer Screening cohort since 2013, which covers major cities and rural areas and facilitates the early detection and treatment of potential lung cancer patients (27). At the same time, medical insurance coverage for cancer treatment has been gradually expanded in Wuhan due to the serious threat of cancer to the health of residents (28). Furthermore, with the adoption of health-related policies such as tobacco control and emission reduction in Wuhan, the rising trend of lung cancer mortality burden has been curbed and started to decline gradually in recent years (29). The cohort effects of lung cancer mortality in the Wuhan population were found to have a turning point around the period when the People’s Republic of China was founded, reflecting that those born in a stable social context could access better medical care or educational resources and have more opportunity to avoid exposure to risk factors related to lung cancer deaths (e.g., smoking, occupational exposure, and poor lifestyles etc.) (30). Also, patients with lung cancer in the same period could be in touch with better treatment after diagnosis and therefore face a lower risk of lung cancer death. Sex was another critical factor affecting lung cancer deaths besides the above factors. The results of this work demonstrated a higher risk of lung cancer deaths in males than in females, which is consistent with previous studies (31, 32). The discrepancy might be attributed to the differences in physiological susceptibilities and behavioral preferences in populations with different sex (33).

Another key finding of our study was that the population’s burden of lung cancer deaths presented a more complex situation in the surrounding urban areas than in the central urban areas in Wuhan. The surrounding urban regions mainly consist of rural areas and large, heavy industrial areas, while the main urban areas include commercial and residential areas. This status might ascribe to the following reasons: First, the medical resources were unevenly distributed in the administrative regions of Wuhan. Because the resources are mainly distributed in the central urban areas, the medical resources allocated in the surrounding urban areas were inferior. They were once even lower than the national average (34). Second, many studies have identified that tobacco exposure was more severe in surrounding urban areas. The epidemic of smoking among adults, tobacco intake among smokers, and secondhand smoke exposure among non-smokers in surrounding urban areas were significantly higher than in central urban areas (35–37).

Furthermore, the heavy industrial areas with more severe air pollution were generally located in surrounding urban areas. Manly considerable cohort research has provided evidence about the relationships between air pollution and lung cancer death, especially in particulate matters (38, 39). A 10 mg/m3 increment in the particulate matter was associated with a 6.2% (PM2.5) and 4.3% (PM10) increase in overall lung cancer mortality, especially among the susceptible population (40). Finally, the gaps in lung cancer mortality between the central and surrounding urban areas of Wuhan might also relate to residents’ education levels, family income, or medical preferences (41). In the city’s future development, the only way to bring the mortality rate of lung cancer under control in the population of Wuhan can only be achieved by addressing the abovementioned issues.

There were some limitations in this study. On the one hand, due to short of the related information about the subtypes of lung cancer subtypes and risk factors of lung cancer in the original data, the analysis of the lung cancer mortality by subtypes and the calculation of risk factors attributable to lung cancer mortality have not been conducted in our study. On the other hand, an ecological fallacy might occur as a type of research based on the population level since this study has paid more attention to the population level rather than the individual level. Thus, subsequent studies should consider the above limitations and make them more complete.

## Conclusion

The burden of lung cancer death in the Wuhan population has shown a gradual decline in recent years, but the impact of aging and population growth on lung cancer mortality should not be ignored. The burden of lung cancer deaths presented a more complex situation in the population of the surrounding urban areas than in the central urban areas in Wuhan. Therefore, the burden of lung cancer deaths in Wuhan might reduce only when the gaps in lung cancer mortality between the central and surrounding urban areas have dwindled.

## Data availability statement

The original contributions presented in the study are included in the article/**Supplementary Material**, further inquiries can be directed to the corresponding authors.

## Author contributions

Conceptualization: CY, YY and NY; methodology: YY and YM; software: YM and YL; validation: YM and YY; formal analysis: YM; resources: CY, YY, XZ, YZ, and NY; data correction: CY, YY, XZ, YZ, and NY; writing-original draft preparation: YM; writing-review and editing: YM and YL; visualization: YM; supervision: CY; project administration: CY, YY. All authors contributed to the article and approved the submitted version.

## Funding

This research was funded by Health commission of Hubei Province scientific research project (Grant No. WJ2019H304), National Natural Science Foundation of China (Grant No. 82173626) and Wuhan Medical Research Project (Grant No. WG20B07).

## Conflict of interest

The authors declare that the research was conducted in the absence of any commercial or financial relationships that could be construed as a potential conflict of interest.

## Publisher’s note

All claims expressed in this article are solely those of the authors and do not necessarily represent those of their affiliated organizations, or those of the publisher, the editors and the reviewers. Any product that may be evaluated in this article, or claim that may be made by its manufacturer, is not guaranteed or endorsed by the publisher.

## Abbreviations

CDC, Disease Control and Prevention; ICD-10, International Statistical Classification of Diseases 10th Revision; ASMRs, age-standardized mortality rates; YLLs, years of life lost;YLLRs, years of life lost rates; ASYLLRs, age-standardized years of life lost rates; ASRs, age-standardized rates; EAPCs, annual percent changes; AAPCs, the average annual percent changes; CIs, confidence intervals; APC, age-period-cohort model; Ad, age-drift model; AP, age-period model; AC, age-cohort model; LDCT, low-dose computed tomography.
