# Supplementary material for: Temporal trends in lung cancer mortality and years of life lost in Wuhan, China, 2010-2019
Source: Front Oncol. 2022 Nov 15;12:1030684. doi: 10.3389/fonc.2022.1030684 (PMC9706208; doi:10.3389/fonc.2022.1030684)
Supplement: Supplementary file 1 [file DataSheet_1.docx]

Supplementary Material

# Detailed information about the Models Used in the Analyses

## The Age-standardized mortality Rates (ASMRs)

The calculation formula was as follows:

$$ASMRs= \frac{\sum_{i}^{A} a_{i}N_{i}}{\sum_{i}^{A} N_{i}}$$

Where $a_{i}$ denotes the age-specific mortality rates at age group $i$. $N_{i}$ is the age-specific population at age group $i$ in the standard population[1].

## The years of life lost (YLLs)

$$YLLs= \sum_{i}^{A} d_{i}e_{i}^{*}$$

Where $d_{i}$ denotes the age-specific death number at age group $i$. $e_{i}^{*}$ means the standardized life expectancy at age group $i$[2].

## The years of life lost rates (YLLRs)

$$YLLRs= \frac{YLLs}{n}$$

Where$n$ is the total number of people in the $i$th age group in Wuhan during the study period[3].

## The age-standardized years of life lost rates (ASYLLRs)

$$ASYLLRs= \frac{\sum_{i}^{j} ({YLLs}_{i}\times\frac{N_{i}}{n}/\frac{n_{i}}{n})}{n}$$

Where $j$ is the life expectancy. $\frac{N_{i}}{n}$ is the population composition ratio of the $i$th age group in the standard population. $\frac{n_{i}}{n}$ is the actual population composition ratio of the $i$th age group in Wuhan[4].

## The estimated annual percent changes (EAPCs) and the average annual percent changes (AAPCs) in Joinpoint model

$$In\left( ASRs \right)= \alpha+\beta_{i}x+\varepsilon$$

$$EAPCs=100\times{(e}^{\beta_{i}}-1)$$

$$AAPCs=100\times(e^{\frac{\sum w_{i}\beta_{i}}{\sum w_{i}}}-1)$$

Where $x$ represents the calendar year. $\beta_{i}$ denotes the annual change. ε means the error term[5].

## The decomposition method

$M_{p}=\sum_{i=1}^{g} {{\left( N_{2}-N_{1} \right)s}_{i1}m}_{i1}$

$$M_{a}=\sum_{i=1}^{g} N_{1}\left( s_{i2}-s_{i1} \right)m_{i1}$$

$$M_{m}=\sum_{i=1}^{g} {N_{1}s}_{i1}\left( m_{i2}-m_{i1} \right)$$

$$I_{pa}=\sum_{i=1}^{g} \left( N_{2}-N_{1} \right)\left( s_{i2}-s_{i1} \right)m_{i1}$$

$I_{pm}=\sum_{i=1}^{g} \left( N_{2}-N_{1} \right)s_{i1}\left( m_{i2}-m_{i1} \right)$

$$I_{am}=\sum_{i=1}^{g} N_{1}\left( s_{i2}-s_{i1} \right)\left( m_{i2}-m_{i1} \right)$$

$I_{pam}=\sum_{i=1}^{g} \left( N_{2}-N_{1} \right)\left( s_{i2}-s_{i1} \right)\left( m_{i2}-m_{i1} \right)$

Where $M_{p}$, $M_{a}$, and $M_{m}$ are the main effects of the changes in population size, age structure, and mortality rate. $I_{pa}$, $I_{pm}$, $I_{am}$, and $I_{pam}$ indicate the two-way and three-way interactions of the effects. The first year in the study period was set as the reference year in the decomposition method.

Chen et.al[6] assumed that the interactions are equally distributed, then the contribution of the three factors can be calculated as follows:

$$A{=M}_{a}+½I_{am}+½I_{pa}+⅓I_{pam}$$

$P{=M}_{p}+½I_{pm}+½I_{pa}+⅓I_{pam}$

$$M{=M}_{m}+½I_{pm}+½I_{am}+⅓I_{pam}$$

$$Net Change=D_{2}-D_{1}$$

Where $A$, $P$, and $M$ represent the contribution of population aging, population growth, and the age-specific mortality rate. $Net Change$ denotes the total change. $D_{1}$ represents the number of deaths of the reference year (2010). $D_{2}$ represents the number of deaths in years other than the reference year (2011 to 2019).

## The age-period-cohort (APC) model

$$\log\left[ \lambda\left( a,p \right) \right]=f\left( a \right)+g\left( p \right)+h(c)$$

Where $\lambda\left( a,p \right)$ denotes the disease mortality at age $a$ and period $p$. $f\left( a \right)$, $g\left( p \right)$, and $h(c)$ represent the age, period, and cohort effects in whole study period[7].

# Supplementary Figures and Tables

## Supplementary Tables

**Supplementary Table S1.** Contribution of changes in population aging, population growth, and age-specific mortality rate of lung cancer to variations of lung cancer deaths in both central and surrounding urban areas of Wuhan, 2010-2019.

| **Year** | **Due to population aging** | **Due to population growth** | **Due to age-specific mortality rate** | **Net change** |
| --- | --- | --- | --- | --- |
| The central urban areas | | | | |
| 2010 (Reference) | -- | -- | -- | -- |
| 2011 | 51.22 | -65.26 | 17.06 | 5.06 |
| 2012 | 93.87 | -57.75 | 99.16 | 139.97 |
| 2013 | 132.86 | -46.39 | 120.81 | 212.04 |
| 2014 | 271.68 | 131.73 | -17.69 | 372.57 |
| 2015 | 357.56 | 166.44 | 38.91 | 539.05 |
| 2016 | 444.40 | 173.69 | 2.56 | 593.35 |
| 2017 | 485.51 | 175.45 | -26.18 | 606.63 |
| 2018 | 584.49 | 266.02 | -43.50 | 759.25 |
| 2019 | 616.07 | 362.83 | -123.23 | 795.72 |
| *Z* values | 3.95 | 3.40 | -1.97 | 3.94 |
| *P* values | **<0.01^*^** | **<0.01^*^** | 0.05^+^ | **<0.01^*^** |
| The surrounding urban areas | | | | |
| 2010 (Reference) | -- | -- | -- | -- |
| 2011 | -2.83 | 5.97 | 113.81 | 116.48 |
| 2012 | 15.15 | 105.12 | 167.52 | 275.56 |
| 2013 | 36.36 | 193.35 | 96.92 | 309.86 |
| 2014 | 145.21 | 14.26 | 371.57 | 527.40 |
| 2015 | 195.77 | 47.71 | 259.12 | 491.45 |
| 2016 | 244.87 | 88.14 | 438.45 | 745.18 |
| 2017 | 275.89 | 97.63 | 315.64 | 662.41 |
| 2018 | 298.28 | 111.60 | 379.33 | 756.19 |
| 2019 | 360.08 | 153.89 | 321.21 | 789.71 |
| *Z* values | 3.76 | 2.14 | 2.33 | 3.57 |
| *P* values | **<0.01^*^** | **0.03^*^** | **0.02^*^** | **<0.01^*^** |

*P* in bold represent statistically significance at *P* < 0.05 (*).

**Supplementary Table S2.** Comparison of age–period–cohort models of lung cancer mortality in Wuhan by sex, 2010-2019.

| **Models** | **Degrees of freedom** | **Deviance** | **Models to compare** | **Degrees of freedom** | **Deviance difference** | ***P* values** |
| --- | --- | --- | --- | --- | --- | --- |
| All population |  |  |  |  |  |  |
| Age (A) | 135 | 446.13 |  |  |  |  |
| Age-drift (Ad) | 134 | 441.91 | Ad vs A | 1 | 4.22 | 0. 42 |
| Age-cohort (AC) | 131 | 261.95 | AC vs A | 3 | 179.96 | **<0.01^*^** |
| Age-period (AP) | 128 | 419.88 | AP vs A | 3 | 172.63 | **<0.01^*^** |
| Age-period-cohort (APC) | 131 | 247.25 | APC vs AC | 3 | 14.70 | **<0.01^*^** |
|  |  |  | APC vs AP | 3 | 22.04 | **<0.01^*^** |
| Males |  |  |  |  |  |  |
| Age (A) | 135 | 339.46 |  |  |  |  |
| Age-drift (Ad) | 134 | 337.32 | Ad vs A | 1 | 2.14 | 0.14 |
| Age-cohort (AC) | 131 | 223.90 | AC vs A | 3 | 113.42 | **<0.01^*^** |
| Age-period (AP) | 128 | 320.26 | AP vs A | 3 | 108.46 | **<0.01^*^** |
| Age-period-cohort (APC) | 131 | 211.80 | APC vs AC | 3 | 12.10 | **<0.01^*^** |
|  |  |  | APC vs AP | 3 | 17.06 | **<0.01^*^** |
| Females |  |  |  |  |  |  |
| Age (A) | 134 | 245.90 |  |  |  |  |
| Age-drift (Ad) | 133 | 243.29 | Ad vs A | 1 | 2.61 | 0.10 |
| Age-cohort (AC) | 130 | 184.93 | AC vs A | 3 | 58.36 | **<0.01^*^** |
| Age-period (AP) | 127 | 238.15 | AP vs A | 3 | 56.22 | **<0.01^*^** |
| Age-period-cohort (APC) | 130 | 218.93 | APC vs AC | 3 | 12.03 | **<0.01^*^** |
|  |  |  | APC vs AP | 3 | 16.15 | **<0.01^*^** |

*P* in bold represent statistically significance at *P* < 0.05 (*).

## Supplementary Figures


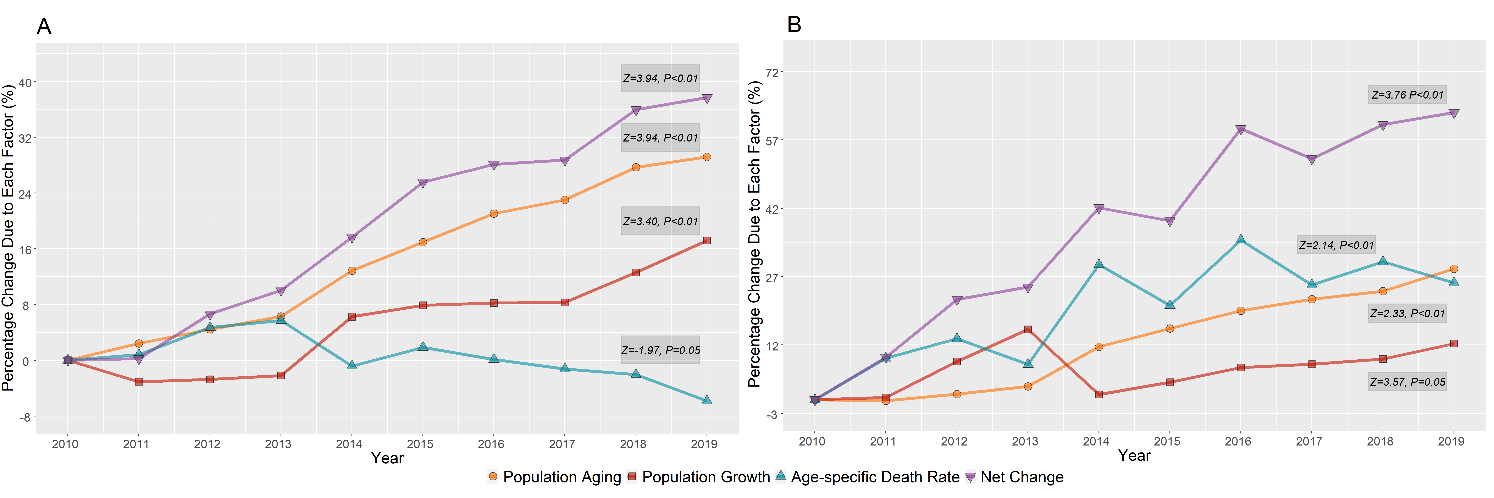


**Supplementary Figure 1.** Relative contributions of changes in population aging, population growth, and age-specific lung cancer mortality rate to variations of lung cancer deaths in both central (A) and surrounding urban areas (B) of Wuhan, 2010-2019.

# Reference

1. Wong MCS, Huang J, Chan PSF, Choi P, Lao XQ, Chan SM, Teoh A, Liang P: **Global Incidence and Mortality of Gastric Cancer, 1980-2018**. *JAMA network open* 2021, **4**(7):e2118457.

2. Kocarnik JM, Compton K, Dean FE, Fu W, Gaw BL, Harvey JD, Henrikson HJ, Lu D, Pennini A, Xu R *et al*: **Cancer Incidence, Mortality, Years of Life Lost, Years Lived With Disability, and Disability-Adjusted Life Years for 29 Cancer Groups From 2010 to 2019: A Systematic Analysis for the Global Burden of Disease Study 2019**. *JAMA oncology* 2022, **8**(3):420-444.

3. Fitzmaurice C, Abate D, Abbasi N, Abbastabar H, Abd-Allah F, Abdel-Rahman O, Abdelalim A, Abdoli A, Abdollahpour I, Abdulle ASM *et al*: **Global, Regional, and National Cancer Incidence, Mortality, Years of Life Lost, Years Lived With Disability, and Disability-Adjusted Life-Years for 29 Cancer Groups, 1990 to 2017: A Systematic Analysis for the Global Burden of Disease Study**. *JAMA oncology* 2019, **5**(12):1749-1768.

4. Yang J, Zhao L, Zhang N, Du Z, Li Y, Li X, Zhao D, Wang J: **Cancer death and potential years of life lost in Feicheng City, China: Trends from 2013 to 2018**. *Medicine* 2021, **100**(39):e27370.

5. Kim HJ, Fay MP, Feuer EJ, Midthune DN: **Permutation tests for joinpoint regression with applications to cancer rates**. *Statistics in medicine* 2000, **19**(3):335-351.

6. Cheng X, Yang Y, Schwebel DC, Liu Z, Li L, Cheng P, Ning P, Hu G: **Population ageing and mortality during 1990-2017: A global decomposition analysis**. *PLoS medicine* 2020, **17**(6):e1003138.

7. McNally RJ, Alexander FE, Staines A, Cartwright RA: **A comparison of three methods of analysis for age-period-cohort models with application to incidence data on non-Hodgkin's lymphoma**. *International journal of epidemiology* 1997, **26**(1):32-46.
